# Long-Term Combinatorial Exposure to Trichloroethylene and Inorganic Arsenic in Genetically Heterogeneous Mice Results in Renal Tubular Damage and Cancer-Associated Molecular Changes

**DOI:** 10.1534/g3.119.400161

**Published:** 2019-03-21

**Authors:** Amie Perry, Rachel M. Lynch, Ivan Rusyn, David W. Threadgill

**Affiliations:** * Department of Veterinary Pathobiology, College of Veterinary Medicine and Biomedical Sciences, Texas A&M University, College Station, TX 77843; † Department of Molecular and Cellular Medicine, College of Medicine, Texas A&M University, College Station, TX 77843; ‡ Department of Veterinary Integrative Bioscience, College of Veterinary Medicine & Biomedical Sciences, Texas A&M University, College Station, TX 77843

**Keywords:** renal toxicity, trichloroethylene, arsenic, environmental exposure

## Abstract

Trichloroethylene (TCE) and inorganic arsenic (iAs) are environmental contaminants that can target the kidney. Chronic exposure to TCE is associated with increased incidence of renal cell carcinoma, while co-exposure to TCE and iAs likely occurs in exposed human populations, such as those near Superfund sites. In order to better understand the kidney health consequences of TCE and/or iAs exposure, a genetically heterogeneous mouse population derived from FVB/NJ and CAST/EiJ mouse strains and deficient for multidrug resistance genes (*Abcb1a^tm1Bor^*, *Abcb1b^tm1Bor^*) was chronically exposed for 52-weeks to varying concentrations of TCE and iAs. Although no exposure group resulted in primary renal cell tumors, kidneys from exposed mice did have significant increases in histologic and biochemical evidence of renal tubular disease with each toxicant alone and with combined exposure, with males having significantly higher levels of damage. Although no added increase in tubular disease was observed with combination exposure compared to single toxicants, molecular changes in kidneys from mice that had the combined exposure were similar to those previous observed in an embryonic stem cell assay for the P81S TCE-induced renal cell carcinoma mutation in the Von Hippel-Lindau syndrome (*VHL*) gene. While this model more accurately reflects human exposure conditions, development of primary renal tumors observed in humans following chronic TCE exposure was not reproduced even after inclusion of genetic heterogeneity and co-carcinogenic iAs.

Trichloroethylene (TCE) is a chlorinated solvent that was widely used as an industrial degreaser and dry-cleaning agent. Due to its wide use and improper disposal, TCE has been found at the majority of the current and proposed National Priority List (Superfund) sites (Scott and Cogliano 2000), and is the most commonly reported organic groundwater contaminant (National Research Council (U.S.) Committee on Human Health Risks of Trichloroethylene. 2006). Exposure to TCE has been associated with a variety of cancers and disorders, including renal cancers and renal injury (Liu *et al.* 2010; Wartenberg *et al.* 2000) and has been associated with a specific mutation in the Von Hippel-Lindau syndrome (*VHL*) gene (Brauch *et al.* 1999). Functional tests of the TCE-associated *VHL* P81S mutation in an embryonic stem cell-based assay showed that it elicits unique characteristics not observed with other *VHL* mutations including diversified cellular metabolism, resistance to apoptosis, and reduced Ataxia-Telangiesctasia mutated (ATM) response to DNA damage (Desimone *et al.* 2013).

There has recently been increased interest and study on the effects of toxicant mixtures on health outcomes (Kapraun *et al.* 2017; Pollock *et al.* 2017), particularly for toxicants like TCE whose effects on disease severity and outcome may be mediated by co-exposures in the environment. Inorganic arsenic (iAs) is a prime candidate for studying interactions of multiple toxicants since it is a commonly encountered environmental contaminant found in soil and groundwater secondary to anthropogenic activities including mining, farming, and fossil fuel combustion (National Toxicology Program 2016; Naujokas *et al.* 2013). Exposure to iAs is associated with adverse health effects in humans including development of chronic kidney disease from chronic exposure to arsenic (Diyabalanage *et al.* 2017; Hsu *et al.* 2017; Zheng *et al.* 2015). In addition to drinking water, iAs exposure may occur through foods and processed beverages such as rice grown in areas with high iAs levels (Naujokas *et al.* 2013; Davis *et al.* 2012; Gilbert-Diamond *et al.* 2011; Jackson *et al.* 2012; Meharg *et al.* 2008). Chronic exposure to iAs at levels higher than the current EPA maximum contaminant level (10 μg/L) are estimated to occur in greater than 3 million people in the United States (Naujokas *et al.* 2013) and it is reasonable to expect that at least some of the people exposed to iAs will also be exposed to other environmental toxicants like TCE.

Co-exposure to potentially carcinogenic toxicants may result in enhancement of carcinogenesis or confound retrospective studies on the effects of a single toxicant. There is increasing evidence that iAs may act as a co-carcinogen or promoter in combination with various other toxicants (Germolec *et al.* 1997; Germolec *et al.* 1998; Rossman 2003; Rossman *et al.* 2001; Rossman *et al.* 2002; Wang *et al.* 2002; IARC Working Group on the Evaluation of Carcinogenic Risks to Humans 2012). Due to the presence of iAs in food and its natural or enhanced presence in the environment, certain areas of the United States have higher exposure levels. This includes areas of North Carolina in the “slate belt” which overlaps with a Superfund site at Camp Lejeune, an area also known to have high levels of TCE contamination (Bove *et al.* 2014; Sanders *et al.* 2012), illustrating the need for research into the potential effects of co-exposure to these toxicants. Onslow County, home to Camp Lejeune had renal cancer incidences of 21.3 per 100,000 from 2008-2012, well above the national average (Centers for Disease Control and Prevention). While studies of exposed human populations provide the strongest link between toxicant exposure and disease, use of appropriate rodent models can provide evidence of toxicity and strengthen the case for human health effects when many epidemiological confounds exists.

The most recent National Toxicology Program (NTP) carcinogenesis study on TCE used a single dose in mice based on the low dose from a preceding 13-week study (National Toxicology Program 1990). Survival of TCE-exposed male mice was lower than that of controls, and cytomegaly was reported in male and female TCE-exposed mice, but none of the vehicle-exposed mice. Increased incidence of hepatocellular tumors was observed in mice, but no renal tumors were observed. While the classic NTP approach has some advantages, it also has substantial limitations. Due to the lack of genetic diversity in the models, these models do not capture the variation in response to toxicants that is due to genetic heterogeneity in humans they seek to model.

In this study, we utilized two common environmental toxicants, TCE and iAs, in doses designed to mirror human exposure levels to model combination environmental exposures, used a genetically heterogeneous population of mice to capture genetic variability, and fed a diet whose nutrient profile is similar to the typical western diet to better model the nutritional environment in which these toxicants would have their effects in the US.

## Materials and Methods

### Animals

All housing conditions and procedures were approved by the Institutional Animal Care and Use Committee. An F3 mouse population was derived from two phylogenetically distant inbred mouse strains. The *Mus musculus domesticus* inbred strain FVB/N-*Abcb1a^tm1Bor^*, *Abcb1b^tm1Bor^* that has mutations in the multi-drug resistance (MDR) transporter genes *Abcb1a* and *Abcb1b*, resulting in loss of function of the MDR transporter. Mice have an especially active MDR system, while *Abcb1a^tm1Bor^*, *Abcb1b^tm1Bor^* double homozygous mutant mice exposed to arsenic have increased sensitivity to acute arsenic toxicity compared to wild-type mice, and higher arsenic accumulation in tissues including the kidney (Liu *et al.* 2002), better modeling that observed in human tissues. The *M. m. castaneus* inbred strain CAST/EiJ is a wild-derived strain that is genetically distinct from the FVB/N strain with a highly divergent polymorphism profile.

Breeding of mice was performed in house at North Carolina State University, Raleigh, NC. The breeding colony and study mice were maintained in a temperature-controlled environment at 21+/− 2° on a 12-hour light: 12-hour dark schedule. Female FVB/N-*Abcb1a^tm1Bor^*, *Abcb1b^tm1Bor^* mice (Taconic Biosciences) were crossed with male CAST/EiJ mice (Jackson Laboratory) to create an F1 population that was intercrossed to create an F2 mouse population. Because only one-quarter of mice in this generation were expected to be *Abcb1a^tm1Bor^*, *Abcb1b^tm1Bor^* double homozygous mutant and a large number of mice with this genotype were required for the study population, F2 mice were genotyped to identify mice homozygous for the double knockout of the MDR transporter as previously reported (Schinkel *et al.* 1997). These mice were then intercrossed to produce the study population of F3 mice homozygous for *Abcb1a^tm1Bor^*, *Abcb1b^tm1Bor^*.

### Toxicant Exposure

One hundred F2 mice (fifty males and fifty females per group) were randomly assigned to each of nine exposure groups for a total of 900 mice in the study population. When assigning mice to each group, only one same sex animal from any given litter was assigned to the same treatment to eliminate litter effects. Study mice were weaned at post-natal day 21 onto AIN-93M standard diet (Envigo-Teklad Diets). At 6-weeks mice were switched from AIN-93M diet onto an American-style diet (Envigo-Teklad Diets) that was designed to be similar to the typical American diet with increased kcal from fat, a skewed omega 6 to omega 3 fatty acid ratio, and deficient for folic acid compared to the AIN-93M standard diet. Mice were allowed to acclimate to the American diet for 10-14 days before being assigned to a specific exposure group. Entry dates into specific treatment cohorts were staggered to minimize confounding by calendar date of procedures, with 200-300 F3 mice entering the study every four to five weeks over a 16-week period. Exposure groups were designed to include no, low, and high doses of each toxicant and all possible combinations of these categories (Table 1). Exposure dosages were calculated as dose equivalents based on human exposure data and low and high doses were selected to be no more than 2 to sixfold different than actual human exposures. For TCE these values included well water measurements of TCE for the high dose and data from the National Health and Nutrition Examination Study (NHANES) for the low dose (National Research Council (U.S.) Committee on Human Health Risks of Trichloroethylene. 2006). Doses for arsenic were based on the World Health Organization (WHO) limit for the low dose and a calculation of arsenic in rice combined with average daily intake for the high dose (Stone 2008). TCE (0, 5, or 2850 ppb) was added to purified drinking water, prepared fresh weekly, and administered in UV-light protected bottles to prevent degradation. iAs (0, 10, or 150 μg/kg as sodium arsenite) was mixed into the cAmerican-style diet. All food was replaced weekly and stored at 4° in vacuum-sealed bags to prevent oxidation. Mice were maintained on their assigned treatment group for the 52-week duration of the toxicant exposure. All sample collections and measurements were performed during narrow time windows to minimize circadian effects.

**Table 1:** Treatment groups

Group	Dose Ratio (iAs:TCE)	iAs Concentration (μg/kg food)	TCE Concentration (ppb in water)
1	None: None	0	0
2	None: Low	0	5
3	None: High	0	2850
4	Low: None	10	0
5	Low: Low	10	5
6	Low: High	10	2850
7	High: None	150	0
8	High: Low	150	5
9	High: High	150	2850

### Sample Collection

Urine was collected prior to the beginning of toxicant exposure, at intermediate times after 19 weeks and 32 weeks of exposure, and prior to euthanasia following 52 weeks of exposure. Mice were individually housed in diuresis cages (Hatteras Instruments Inc., Cary NC) for 16 hr with *ad libitum* access to drinking water. Urine collection tubes were maintained at 1° to 6° for the duration of collection. Urine was centrifuged at 10,000 × g for 5 min to separate particulate matter, 30 μL aliquots were made into fresh clear polypropylene tubes, and aliquots and original sample tube were stored at -80° for future analysis. Samples from the collection just prior to termination were analyzed for this study.

Blood was collected at the same time points as urine. Briefly, blood was collected from the mandibular vein using capillary tubes for collections done prior to and during toxicant exposure. Terminal blood collection was performed by cardiac puncture at the time of necropsy. Blood was allowed to clot for up to 30 min at room temperature in tubes. The blood was then centrifuged at 10,000 × g for 10 min to separate serum from erythrocytes and leukocytes. Serum was transferred into fresh 1.5 mL clear Eppendorf polypropylene tubes and stored at -80° for future analysis. Serum from the terminal collection was analyzed for this study.

Following 52 weeks of toxicant exposure, mice were killed by carbon dioxide asphyxiation followed by cervical dislocation. At necropsy kidneys and other organs (liver, lung, heart, and any organs with abnormal presentations) were removed, weighed, and examined for gross abnormalities. Organ samples were fixed for histology or flash frozen for molecular analysis.

### Histopathologic Examination

Each kidney was halved longitudinally. One half of each kidney was fixed in 10% neutral buffered formalin for 24 hr, transferred to 70% ethanol, then routinely processed and paraffin embedded. Five 5μm-thick serial sections were obtained and the first, third and fifth of these were hematoxylin and eosin (H&E) stained for histopathological examination. Light microscopic examination of kidney slides was performed by a board-certified anatomic pathologist (AP). Each slide was randomly assigned a new identifier to mask exposure group from the pathologist. The first, third and fifth slides for each individual were examined for neoplasia or preneoplastic changes. Following this initial examination, one representative slide from each individual was examined and scored for histologic evidence of renal disease. Only after all samples were scored was the code broken to determine which samples were from each treatment group.

### Measurement of BUN and Creatinine

Measurement of serum blood urea nitrogen (BUN) and creatinine was performed in house using a VetScan VS2 Chemistry Analyzer (Abaxis, Union City, CA) with VetScan Comprehensive Diagnostic Profile Rotors. Serum samples were thawed on ice, mixed with an equal volume of 0.9% saline solution to obtain a final volume of 100 μL, pipetted into the rotor, and analyzed. Creatinine concentrations were below the limits of detection for this instrument for the majority of animals and could not be analyzed.

### Measurement of Urine Protein and Creatinine

Urine protein quantification was performed using a Pierce Coomassie Plus Bradford Assay (Thermo Fisher). A urine sample aliquot was thawed on ice and the assay was performed per manufacturer’s protocol. Briefly, urine samples were diluted with MilliQ water (1:500 for females, 1:1000 for males) and pipetted into microplates. The Coomassie reagent was brought to room temperature before adding to all wells of the plate and incubating at room temperature for 10 min. Samples were measured in duplicate using a microplate reader at 595 nm. Urine protein concentration (mg/dL) was determined based on a standard curve.

Urine creatinine measurement was performed using a Creatinine (urinary) Colorimetric Assay Kit (Cayman Chemical, Ann Arbor, MI). A urine sample aliquot was thawed on ice and the assay was performed according to the manufacturer’s protocol. Briefly, urine samples were diluted with MilliQ water and added in duplicate to the wells of a microplate. Alkaline picrate solution was added to all wells and incubated on a shaker for 10 min at room temperature. Initial absorbance was read at 500 nm. Acid solution was added to all wells and the plate was incubated for an additional 20 min at room temperature before the final absorbance measurement. Samples were measured in duplicate using a microplate reader at 500 nm. Urine creatinine concentration (mg/dL) was determined based on a standard curve.

### Measurement of Urinary NGAL

Urinary Neutrophil Gelatinase Associated Lipocalin (NGAL) measurement was performed using a Mouse Lipocalin-2/NGAL Quantikine ELISA kit (R&D Systems). Urine aliquots were thawed on ice, diluted in the provided assay buffer, and the assay was performed according to the manufacturer’s protocol. Briefly, assay buffer was added to all wells of the kit-provided microplate, samples and standards were added in duplicate, followed by incubation at room temperature. The plate was aspirated and washed, followed by conjugate addition and incubation, substrate incubation in light-protected conditions, and addition of stop buffer. Samples were measured in duplicate using a microplate reader at 540nm/450nm within 30 min of adding the stop buffer. Urine NGAL concentration was determined based on a standard curve. Urine NGAL concentrations were then normalized to urine creatinine and urine osmolality measurements to account for variability in sample concentration.

### Gene Expression Analysis

Frozen kidney samples from low single exposures and low combination exposure groups were thawed and total RNA extracted with an RNAeasy kit (Qiagen). RNA samples were sent to the University of North Carolina Functional Genomics Core for global gene expression analysis with GeneChip Mouse Gene 1.1 ST arrays (Affymetrix, Santa Clara). Gene expression data (CEL files) were analyzed using Partek Genomic Suite, v 6.5 (Partek, St. Louis). Data were normalized by robust multiarray analysis and analysis of variance (ANOVA). Differentially expressed genes were identified between classes using the non-parametric rank-product method (*P* < 0.05), and log_2_ expression values for each gene were generated. Canonical pathway analysis was generated through Ingenuity Pathway Analysis (Qiagen). Functional classes were compared to a previous report (Desimone *et al.* 2013).

### Statistical Analyses

Statistical analysis of Kaplan-Meirer survival curves was performed using a log-rank test in Mstat (Drinkwater 2018). Statistical analyses of histopathologic scoring were performed using JMP 13.0 (SAS, Raleigh, NC) and graphs were built in Prism 8 (GraphPad, San Diego, CA). Tubular disease scores were analyzed by ANOVA with post-hoc testing using the Dunn method for multiple comparisons which utilizes the Bonferroni adjustment to correct for multiple comparisons. The control group for statistical analyses was defined as the untreated group. Biomarkers including BUN, urine protein/creatinine ratio, and urinary NGAL were log transformed to approximate normality, then analyzed by ANOVA with post-hoc testing using the Dunnet method for multiple comparisons with the control group defined as the untreated group.

### Data Availability

All primary data are available in the supplemental file or upon request. Supplemental material available at Figshare: https://doi.org/10.25387/g3.7871525.

## Results

### Survival Is Affected Only by High iAs Exposures

**Figure 1:**
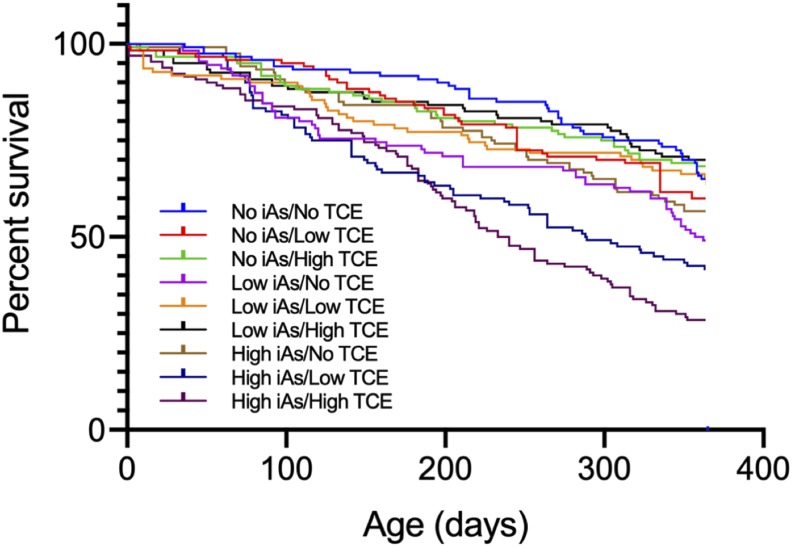
Kaplan-Meier survival plots by dose group.

No significant differences were found in a Kaplan-Meier survival analysis through the end of the study between any dose group lacking High iAs treatment and the No iAs/No TCE (control) group (Figure 1). Log-rank testing of survival showed a significant difference among the groups (*P* < 0.001). Subsequent multiple comparisons showed only the High iAs/Low TCE (*P* = 0.004) and High iAs/High TCE (*P* < 0.001) having higher mortality than the control group, with only the High iAs/High TCE having higher mortality than all other treatment groups except High iAs/Low TCE.

### Toxicant Exposure Is Associated With Renal Tubular Disease

**Figure 2:**
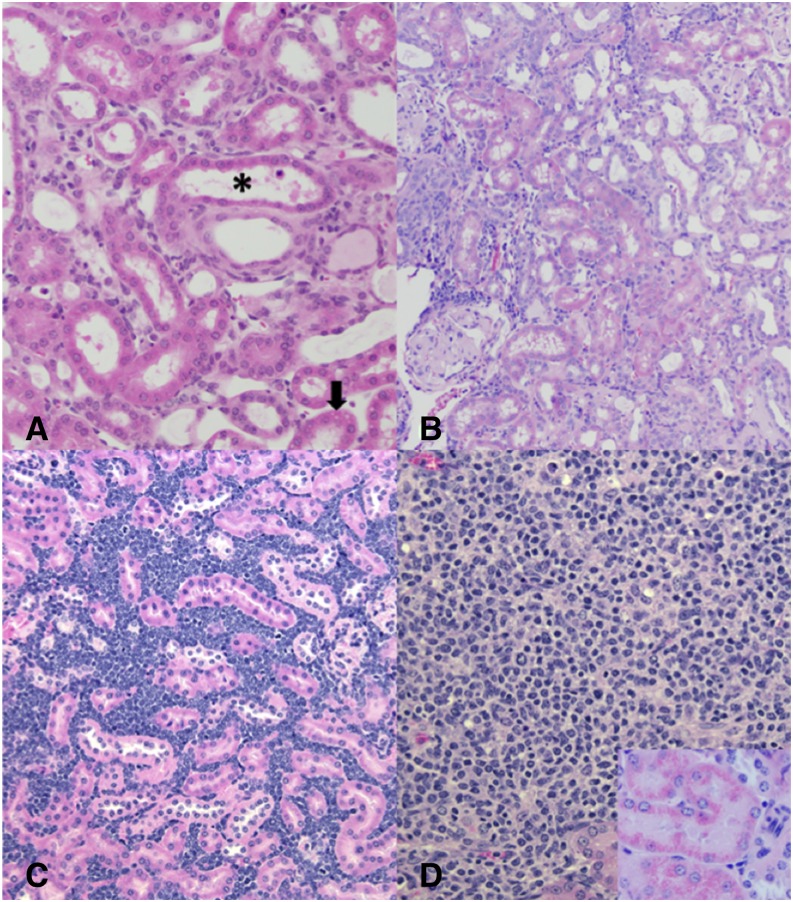
Representative examples of renal pathologies and non-renal tumors. (A) Male. High As/High TCE exposure group. Tubular dilation (asterisk), degeneration and regeneration. One tubule has a necrotic tubular epithelial cell (arrow). (B) Male. No As/Low TCE exposure group. Chronic progressive nephropathy lesions. There are areas of relatively well demarcated tubular change consisting of basophilic tubules with thickened basement membranes. A glomerulus in the section is expanded by presumed amyloidosis. (C) Female. Low As/High TCE exposure group. Lymphoma. (D) Female. High As/High TCE exposure group. Histiocytic sarcoma. Hyaline droplets in renal tubular epithelium support the diagnosis (inset, lower right).

While no changes in overall survival to the end of the study were observed, significant differences between dose groups in tubular disease score as measured by histologic examination were observed. Lesions included in the evaluation were based on numerical criteria scoring (Table 2; Figure 2). Diagnoses were based on criteria published as part of the International Harmonization of Nomenclature and Diagnostic Criteria for Lesions in Rats and Mice (INHAND) Project (Frazier *et al.* 2012). Tubular disease lesion scores were determined by summing the individual scores in each category resulting in a total score for each animal between 0 and 20 (raw scores are in Supplemental Table S1).

**Table 2:** Renal damage scoring matrix

Score	Chronic progressive nephropathy (CPN)	Tubular degeneration +/− regeneration	Tubular dilation	Karyomegaly	Tubular single cell necrosis	Tubular epithelial microvesicles
0	None	None	None	None	Absent	None
1	Rare affected tubules	Rare tubules with evidence of degeneration or regeneration not associated with lesions of CPN	Rare tubules with lumens ≥50% of total tubule diameter	Rare tubular cells with nuclei ≥2X the size of normal nuclei	Present	Present in rare tubules
2	Few foci of affected tubules	Few tubules with evidence of degeneration or regeneration not associated with lesions of CPN	Few tubules with lumens ≥50% of total tubule diameter	Few tubular cells with nuclei ≥2X the size of normal nuclei	N/A	Present in few tubules
3	Many foci of affected tubules	Many tubules with evidence of degeneration or regeneration not associated with lesions of CPN	Many tubules with lumens ≥50% of total tubule diameter	Many tubular cells with nuclei ≥2X the size of normal nuclei	N/A	Present in many tubules
4	Most tubules affected	Most tubules have evidence of degeneration or regeneration not associated with lesions of CPN	Most tubules have lumens ≥50% of total tubule diameter	N/A	N/A	Present in most tubules

**Figure 3:**
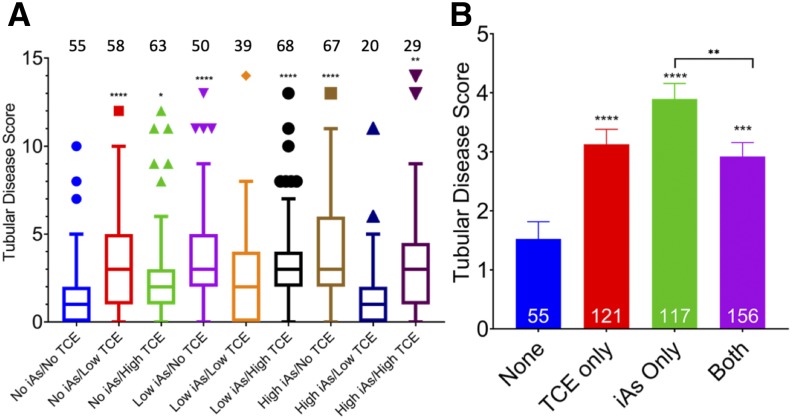
Renal disease scores. (A) Mean (+SE) tubular disease score in each dose group. Number of mice analyzed from each group is shown above. No As/No TCE group was used as control for comparisons: **** *P* < 0.0001; ** *P* = 0.0068; * *P* = 0.0184. (B) Mean (+SE) tubular disease score by toxicant exposure or co-exposure. Number of mice in each group is noted at the bottom. No Toxicant group used as control for comparisons: **** *P* < 0.0001, *** *P* = 0.0003.

Comparisons were made between each treatment group and the unexposed group. Increases in mean tubular disease scores were detected in the No iAs/Low TCE (mean score = 3.47, *P* < 0.0001), No iAs/High TCE (mean score = 2.83, *P* = 0.0184), Low iAs/No TCE (mean score = 3.76, *P* < 0.0001), Low iAs/High TCE (mean score = 3.53, *P* < 0.0001), High iAs/No TCE (mean score = 4.00, *P* < 0.0001), and High iAs/High TCE (mean score = 3.48, *P* < 0.0068) groups as compared to the No iAs/No TCE group (mean score = 1.53) (Figure 3A). In addition, increases in tubular disease scores were observed in animals exposed to TCE alone, iAs alone, and in those exposed to both toxicants in combination, although there was no increase in average severity of disease in those with combination exposure to TCE and iAs compared to those with single toxicant exposure (Figure 3B).

### Males Have Greater Histological Evidence of Renal Disease

Further analysis of the histological tubular disease scoring was performed to investigate the effects of TCE dose, iAs dose, and sex on renal tubular damage. After determining that data were normally distributed, ANOVA analysis showed that sex (*P* = 0.0006) was a significant factor, and that there was a significant interaction between TCE dose and iAs dose (*P* = 0.0005). A Welch’s *t*-test was performed, and, overall, male mice had higher mean total tubular disease scores (mean score 3.678) than female mice (mean score 2.683, *P* < 0.0001).

**Figure 4:**
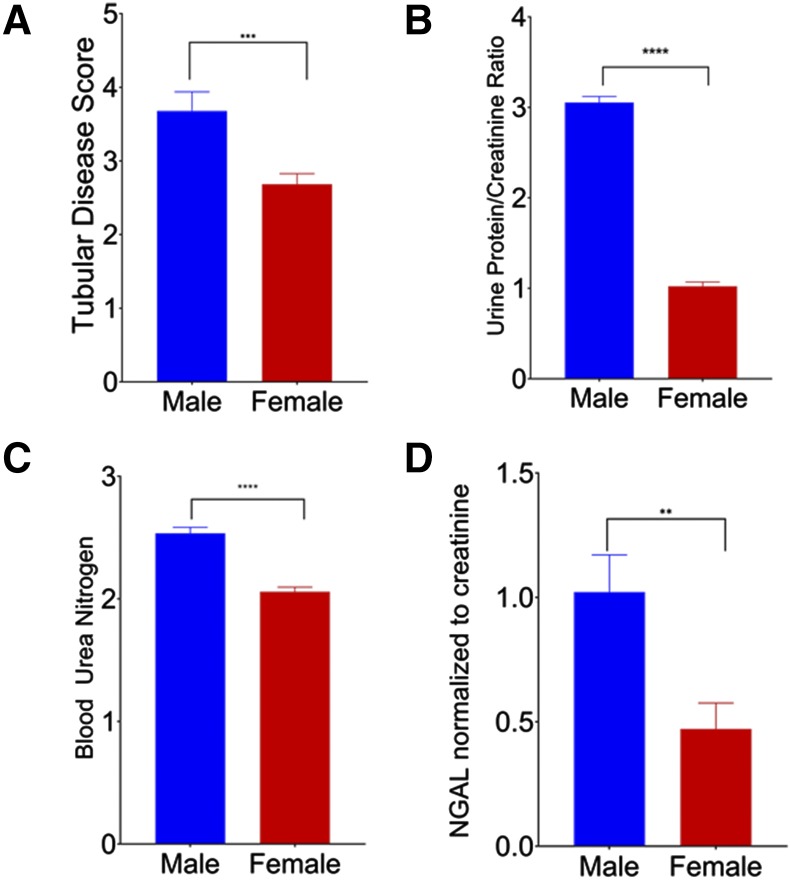
Renal parameters separated by sex. (A) Mean (+SE) tubular damage across all dose groups. Male n = 171; female n = 278 (*P* < 0.0001). (B) Mean (+SE) urine protein/creatinine ratio across all dose groups. Male n = 113, female n = 233 (*P* < 0.0001). (C) Mean (+SE) BUN across all dose groups. Male n = 156, female n = 264 (*P* < 0.0001). (D) Mean (+SE) NGAL normalized to creatinine across all dose groups. Male n = 113, female n = 233 (*P* = 0.0027).

In addition, male mice in general had higher log transformed mean scores for nearly all of the assessed individual renal lesions on histologic examination including: glomerular amyloid/hyaline glomerulopathy (*P* < 0.0001), medullary amyloid or fibrosis (*P* < 0.0001), end-stage kidney (*P* = 0.0034), perivascular cellular infiltrate (*P* = 0.005), chronic interstitial cellular infiltrate (*P* = 0.028), tubular degeneration and regeneration (*P* = 0.0134), tubular single cell necrosis (*P* = 0.0006), pelvic dilation (*P* < 0.0001), infarcts (*P* < 0.0001), and hyaline casts (*P* < 0.0001). This pattern of greater histologic evidence of renal damage held for the biomarkers assessed as well, with UPC, BUN, and urinary NGAL normalized against creatinine all higher in male mice than in female (Figure 4).

### Combination Exposure Does Not Increase Histologic Evidence of Tubular Damage

The highest mean damage score resulted from high iAs and no TCE exposure, but no significant difference was observed between the high iAs/high TCE condition and the high iAs/low TCE condition (Figure 3A). As expected, lowest damage occurred with no toxicants. Interestingly, when the TCE level is high, no significant effects were seen with the addition of iAs exposure. When TCE level is low, higher damage was observed without iAs, and lower damage scores were found when low or high iAs was included. When individual kidney lesion types were examined, those that were more common in toxicant-exposed animals, whether single or combination exposure, compared to the No iAs/No TCE control group were: pelvic cellular infiltrates (*P* = 0.0005), tubular degeneration and regeneration (*P* < 0.0001), tubular dilation in the outer stripe of the outer medulla (OSOM) (*P* = 0.0022), pelvic dilation (*P* = 0.0254), and epithelial vacuolation of tubular epithelium (*P* = 0.0168). Among these lesions, pelvic cellular infiltrates were higher in animals exposed to each single toxicant (TCE only *P* = 0.0064; iAs only *P* = 0.0033) and combination-exposed animals (*P* = 0.0274) as compared to the unexposed group, but there was no difference between combination-exposed animals and single-exposed animals. This pattern held for tubular degeneration and regeneration (TCE only *P* < 0.0001; As only *P* < 0.0001; combination exposure *P* = 0.0011), except that arsenic only exposure resulted in higher tubular degeneration and regeneration score compared to combination-exposed animals (*P* = 0.0004). For tubular dilation of the OSOM, both single exposure to TCE (*P* = 0.0386) and single exposure to iAs (*P* = 0.0003) resulted in higher scores for this parameter compared to those not exposed, but single exposure to iAs resulted in higher scores for this parameter compared to combination exposure (*P* = 0.0100). For tubular vacuolation, only combination exposure resulted in higher scores for this parameter and only in comparison to unexposed animals (*P* = 0.0296).

### BUN Level Correlates With Histology Parameters

Blood urea nitrogen concentrations across the study population were analyzed for correlation to the tubular disease score as well as the urinalysis parameters. BUN values were log transformed before Pearson’s correlation testing was performed. Moderate positive linear correlations were observed between BUN and kidney weight/body weight ratio (0.4268), NGAL normalized to creatinine (0.4349), and NGAL normalized to osmolality (0.3687) (*P* < 0.0001 for all). A slight to moderate positive correlation was observed between BUN and urine protein/creatine (UPC) ratio (0.3237). However, the strongest correlation was found between BUN and tubular disease score (0.4657).

**Figure 5:**
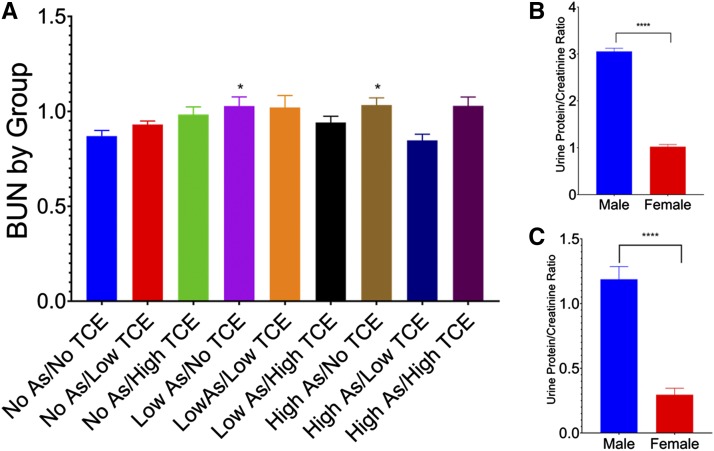
Clinical biomarkers of kidney injury. (A) BUN levels for each exposure group were analyzed and compared to the No iAs/No TCE group. Significance was reached only in the Low iAs/No TCE group (*P* = 0.0137) and the High iAs/No TCE group (*P* = 0.0333) when compared to the No iAs/No TCE group used as the control for this analysis. (B) Urine protein/carnitine ratios for male and female mice across the entire study population. Male n = 113, female n = 233 (*P* < 0.0001). (C) Urine protein/carnitine ratios for male and female mice in the No As/No TCE group. Male n = 12, female n = 31 (*P* < 0.0001).

BUN concentrations for single and combination exposed groups were compared to the No iAs/No TCE group after log transformation of the BUN values to improve normality. ANOVA followed by the post-hoc Tukey-Kramer HSD test indicated that BUN was higher in single iAs exposed animals compared to unexposed animals, but no other differenced were observed. BUN levels for each exposure group were also analyzed and compared to the No iAs/No TCE group using ANOVA with *post hoc* testing using Dunnett’s method. Significance was reached only in the Low iAs/No TCE group (*P* = 0.0137) and the High iAs/No TCE group (*P* = 0.0333) when compared to the No iAs/No TCE group used as the control for this analysis (Figure 5A).

Urinary NGAL was measured and normalized against urinary creatinine. Despite its reported association with tubular damage and slight to moderate correlation with the tubular disease score obtained from histologic examination of the kidneys (0.3395 for NGAL normalized against creatinine and 0.2934 for NGAL normalized against osmolality), no difference was observed in urine NGAL normalized against creatinine (*P* = 0.4443) among the exposure groups, or between single or combination exposed animals compared to the No iAs/No TCE group.

When UPC ratios were compared between the toxicant-exposure groups and the No iAs/No TCE group used as a control, the UPC ratio was higher only in the Low iAs/High TCE group (*P* = 0.0082) and the High iAs/No TCE group (*P* = 0.0145). Male mice across the entire study population had higher UPC values (*P* < 0.0001) (Figure 5B), which was expected given that male mice are known to have higher levels of urinary proteins than female mice. When UPC levels for the entire study population were compared to the UPC levels in the no toxicant control group, the magnitude of the increase was higher among toxicant-exposed mice than in the control population (Figure 5C).

### Exposure and Sex Influence of Non-Renal Neoplasias

No tubular epithelial neoplasms or pre-neoplastic changes of tubular epithelium were observed in the examined sections from any group. However, eight instances of infiltrative round cell neoplasms (histiocytic sarcoma and presumed lymphoma) affecting the kidney were identified (Table 3; Figure 2). Of the eight round cell neoplasms identified in the kidneys, seven were in female mice, and four of the eight were identified in mice with iAs exposure but no TCE exposure.

**Table 3:** Tumor observed in mice surviving to 52-weeks

Dose Group	Sex	Tumor Type	Other Affected Organs
No iAs/No TCE	F	Histiocytic sarcoma	None detected
No iAs/Low TCE	F	Lymphoma	None detected
Low iAs/No TCE	F	Histiocytic sarcoma	Liver, Lung
Low iiAs/No TCE	F	Histiocytic sarcoma	Liver
Low As/No TCE	F	Lymphoma	Liver, Lung
Low iAs/High TCE	F	Lymphoma	Liver
High iAs/No TCE	M	Lymphoma	Liver, Lung, Cranial mediastinal mass
High iAs/High TCE	F	Histiocytic sarcoma	Lung, Lymph node, Cranial mediastinal mass

### Combination Exposure Causes Molecular Changes Similar to That of a TCE-Associated VHL Mutation From Renal Cell Carcinomas

**Figure 6:**
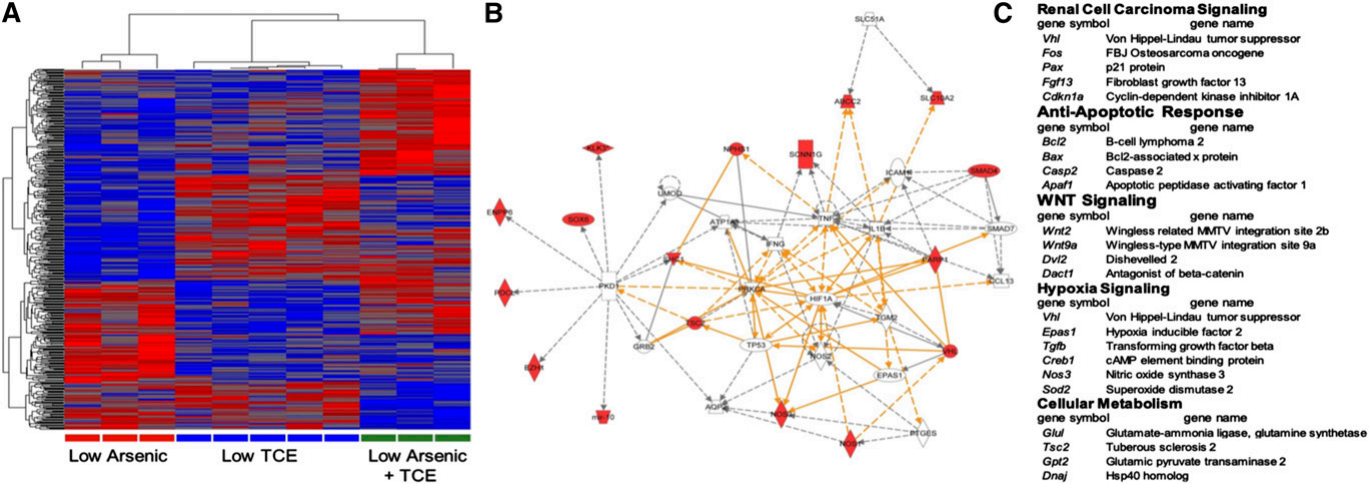
Molecular changes in kidney from exposed mice. (A) Hierarchical clustering of differentially expressed genes. (B) Ingenuity Pathway Analysis of genes differentially expressed in Low iAs/Low TCE exposure groups. (C) Pathway enrichment analysis of genes differentially expressed in Low iAs/Low TCE exposure groups.

Kidney samples from mice that survived 52-weeks of exposure were randomly selected for genome-wide gene expression analysis on Affymetrix Gene 1.1 ST arrays. Hierarchical clustering of all genes that were differentially expressed among the samples revealed that the samples clustered tightly by treatment (Figure 6A). Genes that were differentially expressed between the Low iAs/ Low TCE combination and the two individual treatment groups were over-represented in genes associated with two tumor suppressor genes (*Tsc2* and *Vhl*) known to be involved with human renal cell carcinoma (Figure 6B). Furthermore, pathway enrichment analysis of the Low iAs/Low TCE differentially expressed genes identified the same pathways previously reported for the *VHL* P81S mutation from renal cell carcinomas in people exposed to industrial levels of TCE (Desimone *et al.* 2013). These pathways included those in renal cell carcinoma signaling, anti-apoptotic response, hypoxia signaling, and cellular metabolism.

## Discussion

Developing an accurate rodent model for human TCE-associated renal cell carcinoma has been challenging. Toxicological studies generally evaluate toxicants in isolation and in genetically homogeneous populations of rodents, even though this does not reflect the genetic variability of exposed human populations or the typical scenario of exposure in which multiple toxicants are often encountered together. Response to toxicant exposure is governed by many factors including intrinsic (genetic and epigenetic variation, age and life stage, sex) and extrinsic factors (co-exposures to other toxicants, nutritional state, stressors, dosage, co-morbidities) (Zeise *et al.* 2013).

In this study, we analyzed long-term exposure to TCE alone or in combination with iAs. Previous studies have shown large variability among strains in metabolism and response to TCE (Cichocki *et al.* 2017), suggesting long-term TCE exposure in genetically heterogeneous mice could lead to renal cell carcinoma. In addition to including genetic heterogeneity and nutrition modeled on the typical American diet, our study included a second common environmental toxicant, iAs, to investigate the potential interactions of two commonly occurring toxicants. Arsenic is a known renal toxicant and urinary system carcinogen when exposure occurs through drinking water (Christoforidou *et al.* 2013), and is also known to accumulate and become concentrated in plant-based food products grown in arsenic-containing water (Davis *et al.* 2012; Gilbert-Diamond *et al.* 2011; Jackson *et al.* 2012).

Surprisingly, little differences were observed in survival among dose groups except those on High iAs and any dose of TCE. Even the control group had a relatively large mortality over the 52-week study period, which was almost three-times higher in males than females. One factor contributing to lack of survival differences was conspecific aggression, relevant to control and exposed groups. Aggression among laboratory mice, particularly male mice, is a known issue and has proven difficult to reduce in group housing. Many variables are involved in aggression between laboratory mice, including but not limited to size of housing, number of animals per cage, bedding, shelters, temperature, strain, and stress (Weber *et al.* 2017). Efforts were made in the present study to limit the effects of aggression, such as the use of larger than standard cages and limiting exposure to unfamiliar animals. Even so, some actions that may limit aggression, for example keeping littermates together, could not be implemented in the present study in favor of limiting litter effects.

Although no renal cell carcinomas were observed, significant differences in histologic and biomarker evidence of renal tubular disease were observed among the different treatment groups, including increases in tubular disease scores between animals exposed to TCE alone, exposed to iAs alone, and those exposed to both toxicants, although no difference in severity was observed in those co-exposed, contrary to our expectations. It is possible that damage caused by one toxicant was not increased by exposure to a second renal toxicant as the cells already damaged by the first toxicant, to the point of requiring regeneration, would not be further damaged by the second toxicant. The study was designed to increase renal carcinogenic potential by looking a more diverse genetic backgrounds and adding a second common toxicant. The lack of renal cell carcinomas suggests that these were not limiting factors. Even though renal cell carcinomas were not observed, molecular analysis of kidneys from mice exposed for 52-weeks showed unique gene expression changes associated with combined Low iAs/Low TCE exposure. The changes indicated a kidney cells manifested a tumor promotion state only from the combined exposure that was similar to gene expression changes elicited in an embryonic stem cell assay for the renal cell carcinoma mutation, *VHL* P81S, observed in patients exposed to TCE in an industrial setting (Desimone *et al.* 2013). These results support the link between TCE exposure and molecular changes leading to renal cell carcinoma. Only recently have mice been induced to develop renal cell carcinoma by combining mutations in *Vhl*, *Trp53*, and *Rb1* (Harlander *et al.* 2017). Thus, the reason renal cells in the exposed mice did not progress to cancer is likely due to the fact that *Vhl* mutations alone are not sufficient in to induce renal cell carcinoma in mice.

Further limitations are that the rate of renal cell carcinoma in humans is relatively low and thus the incidence in an animal model might be expected to reflect this. The annual incidence in the United States is 15.6 per 100,000 people (National Cancer Institute). Even in higher incidence areas, such as Camp LeJeune, a TCE-contaminated Superfund site, the incidence rate is only 21.3 per 100,000 people (Centers for Disease Control and Prevention), making the study of these tumors difficult even in a rodent model, due to the large number of individuals required to detect the formation of these tumors. Another factor complicating the modeling of this cancer in rodents is that it requires long periods of time for tumors to develop, increasing the incidence of age-associated sporadic lesions, such as chronic progressive nephropathy (CPN), that complicate histopathologic interpretation. Further, rodents, especially mice, have a higher capacity for metabolism of TCE than humans, (Lash *et al.* 2000), making the toxic metabolite profile and subsequent organ system pathology potentially differ from that seen in humans.

Despite these limitations, the current study used a model more accurately reflecting human exposure conditions by including multiple toxicants, environmentally relevant concentrations, a genetically heterogeneous population, and a long period of exposure. Although a more accurate model of human exposure conditions was used, the primary renal tumor development observed in humans following chronic TCE exposure was not observed in this study. Renal tubular disease did occur both in all treatment groups, and a unique gene expression signature similar to that elicited by expressing a TCE-associated VHL mutation from renal cell carcinomas was observed specifically in the iAs and TCE combined treatment group.
